# Confocal Raman Spectroscopic Imaging for Evaluation of Distribution of Nano-Formulated Hydrophobic Active Cosmetic Ingredients in Hydrophilic Films

**DOI:** 10.3390/molecules26247440

**Published:** 2021-12-08

**Authors:** Louise Van Gheluwe, Emilie Munnier, Hichem Kichou, Kamilia Kemel, Frédéric Mahut, Marylène Vayer, Christophe Sinturel, Hugh J. Byrne, Florent Yvergnaux, Igor Chourpa, Franck Bonnier

**Affiliations:** 1 EA 6295 Nanomédicaments et Nanosondes, Faculté de Pharmacie, Université de Tours, 31 Avenue Monge, 37200 Tours, France; louise.vangheluwe@univ-tours.fr (L.V.G.); emilie.munnier@univ-tours.fr (E.M.); hichem.kichou@univ-tours.fr (H.K.); kami.kemel@gmail.com (K.K.); igor.chourpa@univ-tours.fr (I.C.); 2 UMR CNRS 7374-Université d’Orléans ICMN, 45071 Orléans, France; frederic.mahut@c2n.upsaclay.fr (F.M.); marylene.vayer@univ-orleans.fr (M.V.); christophe.sinturel@univ-orleans.fr (C.S.); 3 FOCAS Research Institute, TU Dublin, City Campus, Kevin Street, Dublin 8, Ireland; hugh.byrne@tudublin.ie; 4 Bioeurope, Route d’Oulins, 28260 Anet, France; florent.yvergnaux@solabia.fr

**Keywords:** active cosmetic ingredients, nanosuspension, polyvinyl alcohol, film, confocal Raman imaging, multivariate analysis, quantitative analysis

## Abstract

Film-forming systems are highly relevant to the topical administration of active ingredients (AI) to the body. Enhanced contact with the skin can increase the efficacy of delivery and penetration during prolonged exposure. However, after the evaporation of volatile solvents to form a thin film, the distribution of the ingredient should remain homogenous in order to ensure the effectiveness of the formula. This is especially critical for the use of hydrophobic molecules that have poor solubility in hydrophilic films. In order to address this concern, hydroxyphenethyl esters (PHE) of *Punica granatum* seed oil were prepared as a nanosuspension stabilised by poloxamers (NanoPHE). NanoPHE was then added to a formulation containing polyvinyl alcohol (PVA) as a film forming agent, Glycerol as a plasticiser and an antimicrobial agent, Sepicide^TM^ HB. Despite their reliability, reference methods such as high-performance liquid chromatography are increasingly challenged due to the need for consumables and solvents, which is contrary to current concerns about green industry in the cosmetics field. Moreover, such methods fail to provide spatially resolved chemical information. In order to investigate the distribution of ingredients in the dried film, Confocal Raman imaging (CRI) coupled to Non-negatively Constrained Least Squares (NCLS) analysis was used. The reconstructed heat maps from a range of films containing systematically varying PHE concentrations highlighted the changes in spectral contribution from each of the ingredients. First, using NCLS scores it was demonstrated that the distributions of PVA, Glycerol, Sepicide^TM^ HB and PHE were homogenous, with respective relative standard deviations (RSD) of 3.33%, 2.48%, 2.72% and 6.27%. Second, the respective relationships between ingredient concentrations in the films and their Raman responses, and the spectral abundance were established. Finally, a model for absolute quantification for PHE was be constructed using the percentage of spectral abundance. The prepared %*w*/*w* concentrations regressed against predicted %*w*/*w* concentrations, displaying high correlation (R^2^ = 0.995), while the Root Mean Squared Error (0.0869% *w*/*w* PHE) confirmed the precision of the analysis. The mean percent relative error of 3.75% indicates the accuracy to which the concentration in dried films could be determined, further supporting the suitability of CRI for analysis of composite solid film matrix. Ultimately, it was demonstrated that nanoformulation of hydrophobic PHE provides homogenous distribution in PVA based film-forming systems independent of the concentration of NanoPHE used in the formula.

## 1. Introduction

The effectiveness of a topical product depends on the physicochemical properties of the active substances and on the capacity of the formula to maintain close contact with the skin for prolonged exposure, enabling the diffusion of the molecules in or through the skin. Topical film-forming systems are increasingly studied for drug delivery because they adhere to the skin, forming a thin transparent film in situ and providing delivery of the active ingredients (AI) to the body tissue [1,2,3]. A cosmetic film-forming system contains three types of components: the active ingredient(s), the excipients (film-forming polymer, plasticizers or additives), and volatile solvent(s) [4] that evaporate upon contact with the skin to form the film.

In 1998, ethyl cellulose (EC) and polyvinyl pyrrolidone (PVP) were used as film formers to develop transdermal drug delivery systems [5]. Since then, various polymers with potential film-forming properties including acrylates polymers (e.g., Eudragit^®^ polymers, Demacryl^®^), cellulose derivatives (Klucel^®^), vinyl polymers (e.g., Kollidon^®^, Polyvinyl alcohol (PVA)), chitosan, starch, and silicones have been studied in this context [6,7,8,9,10]. Plasticisers can be added to formulations to render them appropriately flexible for use on the skin and to increase drug diffusion [7]. Additionally, some chemical enhancers can be added to polymeric film formulations in order to improve the skin penetration of active ingredients [11].

The resulting film should form a homogenous reservoir of the active ingredient on the skin. It has been reported in the literature that only drugs in molecular (as opposed to solid) form in the residual film can diffuse [4]. During the film-forming process, the evaporation of volatile components can change the characteristics of the formulation and even produce drug precipitation on the skin surface (so called vehicle metamorphosis [12]). This phenomenon is particularly observed for hydrophobic AI dispersed in a hydrophilic film. To prevent the precipitation of AI, effective precipitation inhibitors [13] can be added to the mixture, or the hydrophobic AI can be encapsulated [14]. 

Physicochemical characterisation of newly developed products can be a pivotal step in optimising and refine formulae to reach the desired texture and sensorial properties without loss of efficacy. Commonly, topical films are characterised in terms of their morphology and mechanical properties. For example, surface texture (smooth or rough) of the film can be observed using light microscopy, scanning electron microscopy (SEM) [15,16,17,18] and atomic force microscopy (AFM) [19,20,21]. Analytical control to confirm the concentration of ingredients in films can be performed with gold standard techniques such as high-performance liquid chromatography (HPLC) [17]. However, to determine the content of an AI, fastidious extraction protocols need to be applied prior to analysis. Firstly, such approaches fail to deliver spatially resolved information about AI distribution in the films. Secondly, there is an increasingly high demand from the cosmetic industry for greener methods that are environmentally responsible, and hence alternatives with lower requirements of consumables and solvents are sought.

Vibrational spectroscopy, i.e., infrared absorption and Raman scattering spectroscopy, is a non-destructive, label-free and reagent (solvent)-free technique that provides molecular fingerprints, enabling the chemical composition of samples to be probed. Fourier transform infrared spectroscopy (FTIR) has also been used for the characterisation of films [1,15] and or to identify the molecular interactions between active ingredients and the other constituent components of films [16,19]. Use of Raman spectroscopy has been reported for study the composition of various thin polymer films [14,20,21]. Most studies have relied on point measurements [1,15,19,20,21], whereas coupling the molecular specificity of the analysis with the micrometric spatial resolution of Raman confocal microscope offers a powerful non-destructive tool to study the local distribution of AI in cosmetic films. Confocal Raman Imaging (CRI) has been extensively studied in, for example, the sub-cellular analysis of single cells to investigate physiological processes [22] or the internalization of anticancer drugs [23] and nanoparticles [24] as well as the cartography of tissue sections from different organs for diagnostic purposes [25], monitoring AI penetration into the skin [26] and the analysis of solid forms such as pharmaceutical tablets for quality control [27] and identifying counterfeits [28].

More recently, a study using Raman cartography highlighted that hydrophobic compounds from *Punica granatum* seed oil hydroxyphenethyl esters (PHE) stabilised in a poloxamer-based nanosystem showed very homogeneous distribution in hydrophilic PVA films compared to the free form introduced as an ethanol solution [14]. While quantitative analysis of AI concentrations in cosmetic and pharmaceutical products using Raman spectroscopy is increasingly documented for solutions and semi-solids forms (gels, cream) [29,30,31], application of the technique to solid complex mixtures remains largely limited to semi-quantitative analysis [32,33,34].

In the current study, Raman spectroscopy coupled with Non-negatively Constrained Least Squares (NCLS) analysis is used to deliver reconstructed chemical maps to evaluate not only the distribution but also the constituent chemical content of nanoformulated *Punica granatum* seed oil hydroxyphenethyl esters (NanoPHE) in thin hydrophilic PVA films containing Glycerol as a plasticiser along with an antimicrobial agent. Increasing concentrations of NanoPHE were incorporated in PVA-based films to study the homogeneity of the AI distribution and evaluate the compatibility of the formula with a broad range of concentrations. Moreover, achieving quantification of the ingredients using Confocal Raman Imaging (CRI) is addressed, while the technique is proposed as a non-destructive and reagent-free approach that can support the shift towards green chemistry and environmentally responsible industrial processes in the cosmetic field.

## 2. Materials and Methods

### 2.1. Materials

*Punica granatum* seed oil hydroxyphenethyl esters (PHE), commercialized as Delipidol^®^, were kindly provided by BioEurope (Solabia group, Anet, France). Pluronic^®^ F127 was kindly provided by BASF (Levallois-Perret, France). Polyvinyl alcohol (Mw: 31,000–50,000 g/mol, 87–89% hydrolysed) (PVA), Vitamin E (VitE) and dimethyl carbonate (DMC) were purchased from Sigma-Aldrich (Saint-Quentin-Fallavier, France). Glycerol was purchased from Cooper^®^ (Melun, France) and Sepicide^TM^ HB from SEPPIC^®^ (La garenne-Colombes, France). Sepicide^TM^ HB is a mixture of phenoxyethanol, methylparaben, ethylparaben, propylparaben and butylparaben, henceforth referred as AMA (for AntiMicrobial Agent) in the text. Ultrapure water was obtained with a MilliQ system (Millipore, Molsheim, France).

### 2.2. Methods

#### 2.2.1. Sample Preparation

**Nanoformulation of PHE (NanoPHE:** NanoPHE were prepared by a green emulsion–solvent evaporation process assisted by ultrasound, as described in a previous study [14]. Briefly, the aqueous phase was prepared by dissolving 600 mg of Pluronic^®^ F127 in 10.5 g of ultrapure water. The organic phase was prepared by dissolving 1225 mg of DL and 80 mg of Vitamin E in 2.70 g of dimethyl carbonate. The nanoemulsion was obtained by sonicating the mixture during 2 min in a glass rosette cell (20 kHz, amplitude 30%, Vibracell 75041-Sonics). The organic solvent was evaporated (60 mbar 1 h, 25 °C, LABOROTA 40001-Heidolph) to obtain nanodispersion (NanoPHE). The volume of the preparation was adjusted to 20 mL with ultrapure water to ensure the reproducibility of the concentration. 

**Characterisation of NanoPHE:** The hydrodynamic diameter (D_H_) and polydispersity index (PdI) of NanoPHE were measured at 25 °C using a dynamic light scattering (DLS) instrument (NanoZS, Malvern Instruments, Orsay, France) piloted by Zetasizer Software (zetasizer software.ink, version 7.10, Malvern panalytical, Worcester, UK). Each sample was diluted 1:10 in ultrapure water, then measured using a 633 nm laser with a detection angle of 173°. NanoPHE zeta potential was measured at the same dilution and with the same equipment, with a detection angle of 13°. All measurements were carried out in triplicate.

**Figure 1:**
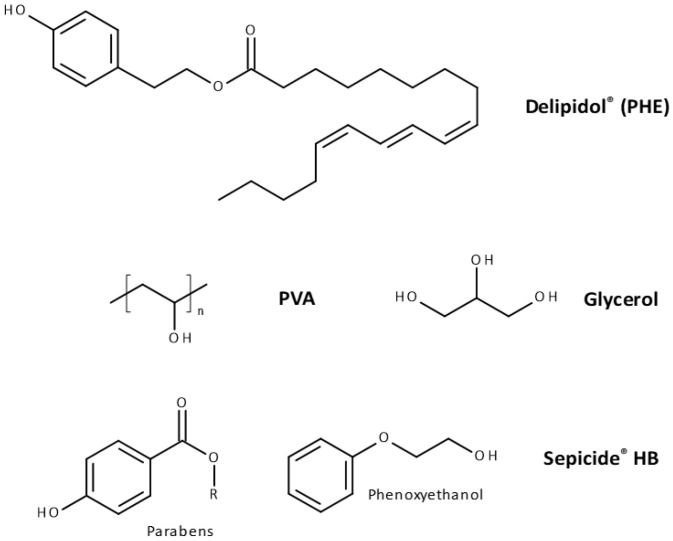
Main components for the preparation of PVA-GS films.

**PVA-GS NanoPHE-loadedfilm preparation:** Six different film-forming solutions were prepared within a constant matrix: PVA, Glycerol and AMA (abbreviated as PVA-GS), with different concentration of NanoPHE (abbreviated as C0, C1, C2, C3, C4, and C5). Figure 1 shows the chemical formulae of these compounds.

PVA solution was prepared under heating (90 °C) and vigorous stirring until complete dissolution in ultrapure water (approximately 4 h), then cooled to room temperature. In order to avoid potential degradation of other components, PVA solutions were prepared upstream, then Glycerol, AMA, and NanoPHE were added. Glycerol and AMA were first added to the PVA solution and stirred for 20 min; finally, the NanoPHE suspension was added and stirred for 30 min before film preparation.

For each sample, 10 g of the final film-forming solution was prepared (Table 1). The initial PVA solution was adjusted for C0–C5 to keep the final concentration at ~10% *w*/*w*. Glycerol and AMA had respective final concentrations of ~10% *w*/*w* and 1% *w*/*w*. NanoPHE concentration ranged from 1.15% *w*/*w* (C1) to 10.218% *w*/*w* (C5). The final nominal concentration (%*w*/*w*) for each component in the dried films is provided in Table 2.

Films were prepared on Superfrost^®^ histological glass slides (ThermoScientific, Waltham, MA, USA; Menzel-Gläser, Braunschweig, Germany) by scraping the solution between four adhesive tapes used to form a rectangular shaped mould. Another clean glass slide was used to spread the sample over the entire length of the substrate, leaving a liquid film with a constant thickness. Samples were left to air-dry overnight in darkness. Films for each concentration were prepared in triplicate and identified as R1, R2 and R3.

#### 2.2.2. Confocal Raman Imaging

**Acquisition:** Raman images were acquired using an Alpha 300 R microspectrometer (WITec, Ulm, Germany). Spectral data were recorded using a 532 nm excitation laser source with a power of 20 mW at the sample. The instrument is calibrated daily using a two-step procedure. First, the TrueCal function of Project 5 (WITec, Ulm, Germany) was used; this is an automatic multipoint calibration routine performed with a Mercury-Argon (HgAr) light source integrated in the Raman microscope. Second, prior to data acquisition, verification was done using the peak at 520.7 cm^−1^ from a silicon substrate. The laser was focused onto the sample through a 50× objective (Zeiss, LD Epiplan HD, N.A. 0.5). Backscattered light was detected by dispersing Raman-shifted radiation onto a Deep Depletion CCD detector (1024 × 128 pixels) using a 600 lines/mm grating centred at 2500 cm^−1^, resulting in a spectral resolution of ≈4 cm^−1^. The acquisition time was set to 10 s, with two accumulations. 100 × 100 µm^2^ Raman maps were recorded with an X and Y lateral step size of 4 µm (corresponding to 625 spectra). For each concentration and condition, three separate films were prepared (R1, R2, R3), and the measurements were performed once for each sample. Additionally, reference Raman spectra were recorded for all constituent compounds of the films.

**Data handling:** Raman maps were pre-processed and analysed using Matlab^®^ (Mathworks, Portola Valley, CA, USA). Spectra were cut in the range 300 to 1800 cm^−1^ and then subjected to offset correction followed by vector normalisation.

**Principal Components Analysis (PCA):** PCA is a multivariate analysis technique that is widely used to simplify a complex data set of multiple dimensions. It permits a reduction in the number of variables in a multidimensional data set while retaining most of the variation within the data set. The other advantage of this method is the derivation of component (PC) loadings, which represent the variance of each variable (wavenumber) for a given PC. Analysis of the loading vector of a PC can provide information about the source of the variability inside a data set derived from variations in the chemical components contributing to the spectra [26].

**Non-negatively Constrained Least Squares analysis (NCLS):** NCLS is a method for the unmixing of spectral data described by Kwan et al. [35]. The NCLS algorithm calculates the signal contribution of the molecule of interest in each experimental Raman spectrum collected from the sample based on a set of reference spectra. A simplified description of the NCLS outcome can be defined as Equation (1):

S_S_ = (F_1_ × S_R1_) + (F_2_ × S_R2_) + + (F_i_ × S_Ri_) + R (1)

where S_S_—simulated spectrum for a given pixel of the Raman map, S_Ri_—reference spectra, F_i_—abundance fraction estimated, i—number of reference spectra included in the model and R—the residual. The algorithm aims to calculate an S_S_ which is as close as possible to the experiential spectrum, with minimal residual.

The NCLS algorithm requires reference spectra in order to calculate the relative spectral contributions of each compound for every pixel of the Raman map. Calculation cannot be performed with a single reference spectrum [26], and it has been demonstrated by Miloudi et al. that increasing the number of replicates for a given reference compound significantly increases the reliability of the outcome [26]. Therefore, for each component (free or encapsulated PHE, Vitamin E, Pluronic^®^ F127, Glycerol, and AMA), ten spectra were used in the NCLS. Concerning the PVA, a control film containing only the polymer was prepared and a total of 1875 reference spectra were collected and used in the NCLS. For clarity, the results are presented as the mean contribution of each compound, calculated from the n replicates of reference spectra.

First, the results were presented as reconstructed heat maps, with a scale bar ranging from blue (low concentration) to red (high concentration) reflecting the spectral contribution calculated from reference spectra, as determined by NCLS fitting. Individual maps were therefore obtained for each individual reference compound used for the NCLS calculations. The obtained NCLS score corresponds to the spectral abundance fraction of a given ingredient, and it is between 0 and 1. A value of 1 is equal to 100% of the reference spectrum used in the calculation.

Second, NCLS results were expressed as the percentage of spectral abundance. The contribution F of a given compound was normalised to 1 by dividing its score obtained by NCLS by the sum of the scores of all references used, then multiplied by 100, as follows:

(2) $$\%\;\mathrm{spectral}\;\mathrm{abundance}\;\mathrm{of}\;X\;\mathrm{in}\;\mathrm{film}\;C_{n}=\frac{F_{x}}{\left(F_{1}+{\;F}_{2}+\cdots +{\;F}_{i}\right)}\times 100$$

where X—PVA, Glycerol, AMA or PHE, C*n*—the concentration of NanoPHE in the film, *n*—the number of concentrations tested (from 0 to 5), F—the spectral abundance fraction estimated from NCLS, i—the number of reference compounds included in the model. The spectral abundance was then used to construct linear regression plots.

It should be noted, however, that while the terms spectral abundance and percent spectral abundance are used in the description above, each constituent compound has a different Raman cross-section, and therefore can contribute more or less strongly to the Raman spectrum of the film. The percentage-based spectral maps thus provide a semi-quantitative representation of the relative contributions, but do not directly correspond to the actual percentages of the concentrations. 

Thirdly, in an effort to take the final step towards full quantification, a calibration model for PHE is constructed using the percentage of spectral abundance as a function of %*w*/*w* PHE in dried films. The results for maps R1 of each concentration (C0 to C5) were used as data points for the calibration curve, then the percentages of spectral abundances calculated from the maps R2 and R3 were projected in the model as unknown samples to be determined. A linear regression of the predicted concentration against the prepared concentrations resulted, with the linearity (coefficient of determination-R^2^) and Root Mean Square Error (RMSE) used as criteria of the model’s reliability, with RMSE defined as follows:

(3) $$\mathrm{RMSE}=\sqrt{\overset{n}{\underset{i=1}{\sum }}\frac{{\left({ŷ}_{i}-y_{i}\right)}^{2}}{n}}$$

where *n*—number of observations, y_i_—observed values, and ŷ_i_—predicted values.

## 3. Results and Discussion

### 3.1. Preparation and Characterisation of NanoPHE

Pluronic^®^ F127 is a non-ionic amphiphilic triblock polymer, poly(ethylene oxide)_101_–poly(propylene oxide)_56_–poly(ethylene oxide)_101_. Composed of two hydrophilic blocks (polyethylene oxide, PEO) and one hydrophobic (polypropylene oxide, PPO), the micellar organisation of Pluronic F127 in aqueous solution induces a core-shell nanostructure. In this study, the hydrophobic active ingredient PHE has been housed in the polypropylene oxide hydrophobic core, producing NanoPHE structures.

The nanoformulation size was characterized by DLS. NanoPHE aqueous suspensions typically displayed hydrodynamic diameter values of 126.3 ± 1.0 nm and exhibited low PdI values (0.107 ± 0.002), indicative of the monodispersity of the suspension. The zeta potential was slightly negative. Electrostatic repulsion of the particles was not an issue, as the colloidal stability was expected to be ensured by the steric hindrance of the PEO chains of Pluronic^®^ F127, stabilising the system [36]. 

The properties of NanoPHE were similar to those previously reported, highlighting the reproducibility of the protocols [14]. These nanostructures, with a D_H_ close to 120 nm, can be considered as suitable for skin administration [37].

### 3.2. Spectral Characterisation of NanoPHE

**Figure 2:**
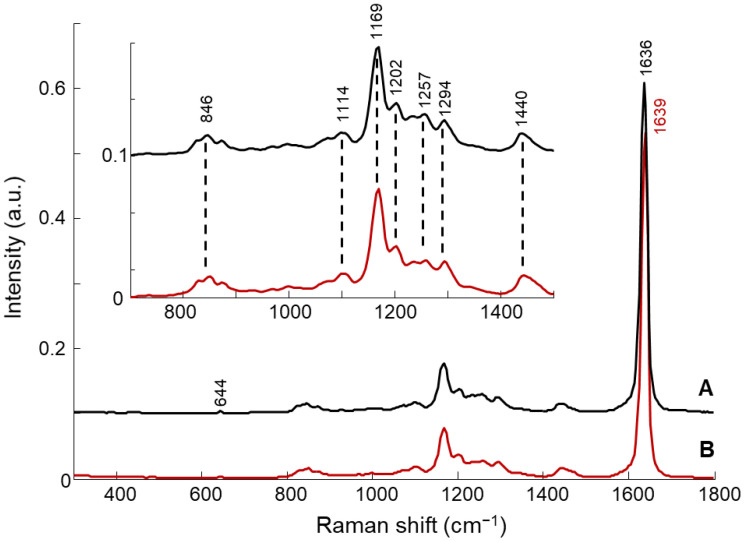
Typical Raman spectra of free PHE (**A**) and nanoformulated PHE (NanoPHE, **B**). Spectra are offset for clarity.

The Raman spectrum of PHE (Figure 2A) exhibits two strong features, at 1636 cm^−1^, corresponding to C=C stretching, and at 1169 cm^−1^, corresponding to C-C and C-O stretching. Other bands in the range of 800 cm^−1^–1450 cm^−1^ display lower intensities, but can still be easily observed in the enlarged inset of Figure 2. The corresponding vibrational mode assignments are listed in Table 3.

The Raman spectrum of NanoPHE (Figure 2B) is similar to that of pure PHE, with a significant band shift in the C=C stretching of PHE from 1636 cm^−1^ to 1639 cm^−1^, explained by molecular interaction with other components from the shell or the core of the nanosystem. The other features do not appear to be modified by the nanoformulation.

**Figure 3:**
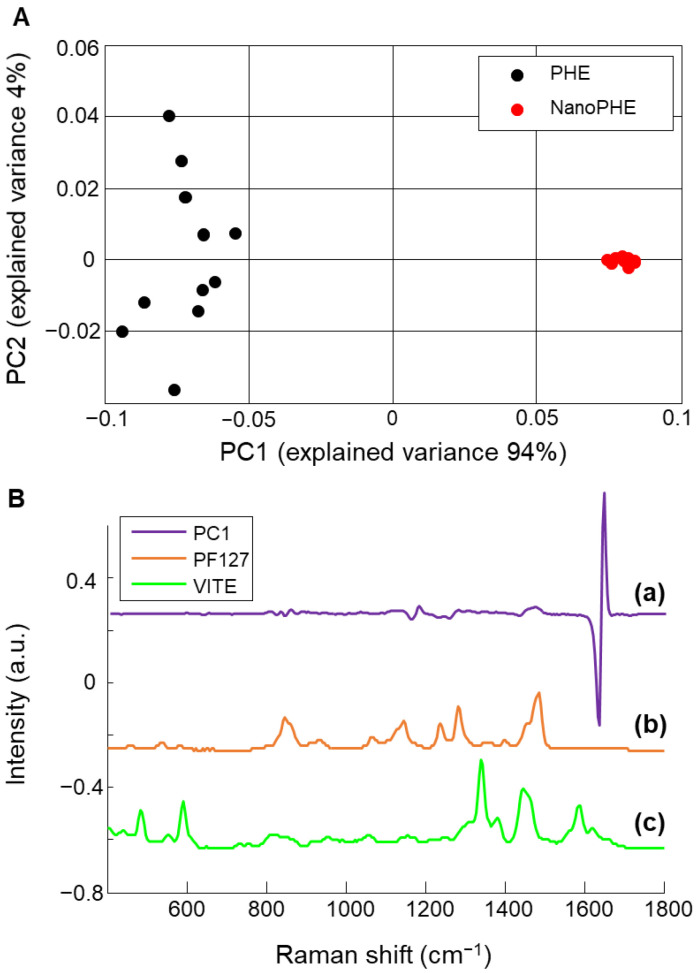
(**A**): PCA Scatter plot from reference spectra of free (black) or NanoPHE (red). (**B**): Loading 1 from the PCA (**a**) compared to the reference spectra of Pluronic^®^ F127 (**b**) and vitamin E (**c**). Spectra are offset for clarity.

The PCA scatter plot in Figure 3A shows a clear separation along Principal Component 1 (PC-1) (94% of the total explained variance) between the data corresponding to PHE spectra (black dots) and NanoPHE spectra (red dots). The loading plot corresponding to PC-1 (Figure 3(Ba)) shows that the main features driving the discrimination between PHE and NanoPHE are the 1636–1639 cm^−1^ peaks, corresponding to C=C stretching of the aromatic ring of PHE.

However, there is no clear evidence that the Pluronic^®^ F127 (Figure 3(Bb)) found in the shell of nanosystems or the VitE (Figure 3(Bc)) present in the core mixed with PHE contributed significantly to the collected spectral signature. The main features of Pluronic^®^ F127 [38] (Figure 3(Bb)), observed at 844–860 cm^−1^ (methylene and methyl rocking), 1144 cm^−1^ (C-C and C-O stretching modes), 1380 cm^−1^ (CH_3_ twisting) and 1450 cm^−1^ (CH_2_ scissoring), do not appear in the PC1 loading. Similarly, the bands specific to VitE [39,40] (Figure 3(Bc)) at 490 cm^−1^ (ring structure), 591 cm^−1^ (O-H out of plane bending), 1340 cm^−1^ (CH_2_ twisting), 1450 cm^−1^ (CH_2_ scissoring), 1582 cm^−1^ (C=C stretching aromatic ring) and 1615 cm^−1^ (C=C stretching aromatic ring) are not observed.

### 3.3. Spectral Characterisation of PVA-GS-NanoPHE Films

#### 3.3.1. Reference Compounds

**Figure 4:**
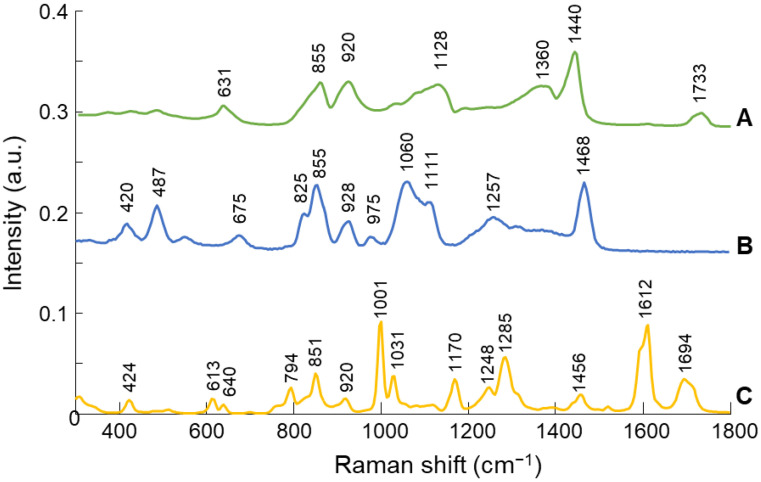
Raman spectra of other main compounds of PVA-GS-NanoPHE films: (**A**) control PVA film, (**B**) Glycerol, and (**C**) AMA. Spectra are offset for clarity.

Reference Raman spectra of NanoPHE, PVA, Glycerol and AMA were recorded to identify the characteristic vibrational bands of the molecules (Figure 4).

The main spectral features of PVA (Figure 4A) occur at 855 cm^−1^ (C-C stretching), 920 cm^−1^ (C-C stretching), 1128 cm^−1^ (C-O stretching, O-H bending), 1360 cm^−1^ (C-H and O-H bending), and 1440 cm^−1^ (C-H and O-H bending) [41]. The Raman spectrum of Glycerol (Figure 4B), in the 1000–1500 cm^−1^ spectral region, contains two broad bands that encompass modes due to CCO stretching, CH_2_ twisting, and COH stretching vibrations of the primary -H groups. The bands at 1060 and 1111 cm^−1^ are assigned to the symmetric stretching mode of the primary and secondary -OH groups, respectively. The bands in the 1207–1310 cm^−1^ spectral region and the band at 1468 cm^−1^ are assigned to CH_2_ twisting [42].

The Raman spectrum of AMA (Figure 4C) contains an intense Raman line at 1001 cm^−1^, corresponding to the aromatic ring of its components, phenoxyethanol and parabens [43,44]. Further intense features were observed at 1285 cm^−1^ (CH_2_ twisting, C-H (ring) bends, and aromatic ring deformation of phenoxyethanol), 1612 cm^−1^ (C-H (ring) bends and aromatic ring deformation of phenoxyethanol) and 1694 cm^−1^ (C=O stretching of parabens) [43,44].

#### 3.3.2. PVA-GS-NanoPHE Films

**Figure 5:**
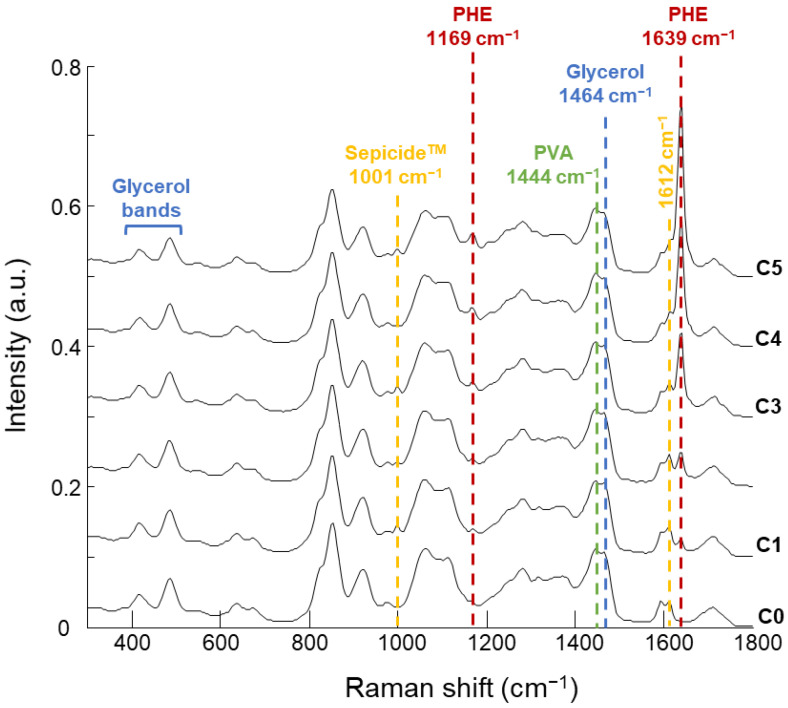
Mean Raman spectra of PVA-GS films loaded with increasing concentration (from C0 to C5) of NanoPHE. Spectra offset for clarity. Dotted line highlights most relevant features of PVA (green), Glycerol (blue), AMA (yellow), and PHE (red).

Each pixel from a Raman map contains a full spectrum resulting from the combined contribution of all constituents. Figure 5 displays the mean spectra obtained from PVA-GS-NanoPHE films with increasing concentrations of NanoPHE (C0 to C5).

The mean spectrum of C0 exhibits the spectral features of the films formed from the mixture without NanoPHE. The signature is mainly due to the combined contribution of PVA and Glycerol, most of whose peaks overlap, for example in the spectral ranges 825–855 cm^−1^, 920–928 cm^−1^, 1060–1128 cm^−1^, and 1440–1468 cm^−1^. The contribution from AMA is limited to the 1001 cm^−1^ and 1612 cm^−1^ features.

**Figure 6:**
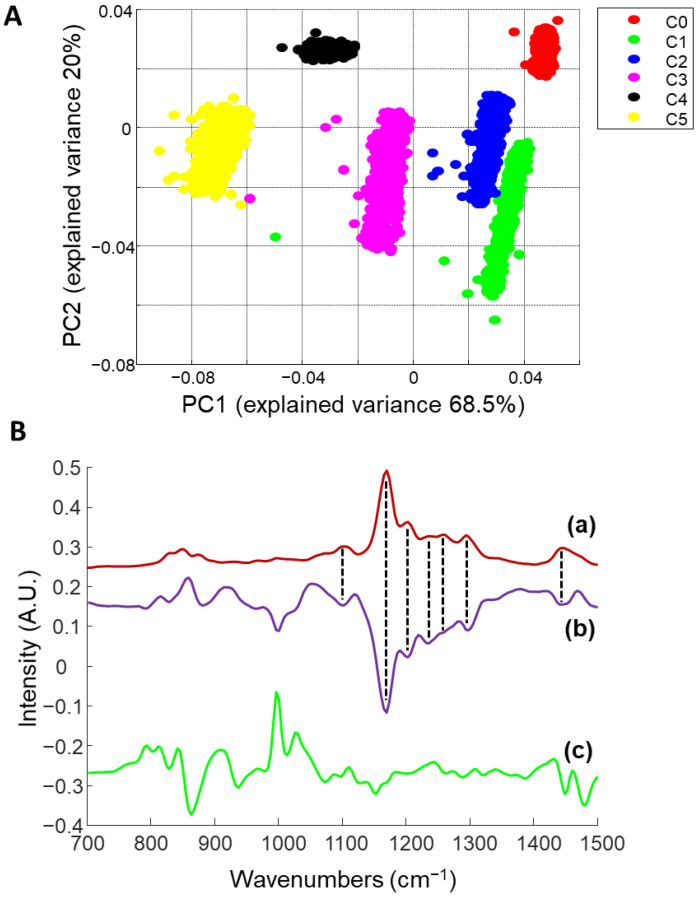
(**A**): Scatter plot from principal component analysis (PCA) performed on PVA-GS films with increasing NanoPHE concentrations (C0: red, C1: green, C2: blue, 3: pink, C4: black, and C5: yellow). (**B**): Loading 1 (**b**) and Loading 2 (**c**) of the PCA (**b**) compared to the reference spectra of NanoPHE (**a**). Spectra are offset for clarity.

The emergence of the strong PHE feature at 1639 cm^−1^ as a function of NanoPHE concentrations added to the films is particularly noticeable (Figure 5, C1–C5). At first, the PHE band appears as a separate band beside features from AMA (Figure 5, C1 and C2) but for higher concentrations, the dominance of the PHE band results in a merging with the weaker AMA features (Figure 5, C3, C4 and C5). Over the range of 300–1500 cm^−1^, the spectral variations linked to PHE are not as obvious. However, PCA performed in the 700 and 1500 cm^−1^ region leads to a clear separation of data clustered as a function of PHE concentrations along the principal component 1 axis (PC1, 68.5% of the total explained variance) (Figure 6A).

Interestingly, the loading plot (Figure 6(Bb)) highlights spectral differences matching features specific to PHE (Figure 6(Ba)), which are negative, consistent with the increasing PHE concentration (Figure 6A). The negative peak at 1001 cm^−1^ cannot be assigned to PHE but indicates the contributions of AMA (See Figure 5) to the composite spectrum. Positive peaks between 800–950 cm^−1^ are PVA- and Glycerol-related. It can be observed on the scatter plot that PC 2 accounts for 20% of the explained variance. The PC 2 loading vector is presented in Figure 6(Bc). There is no evidence of any positive or negative features linked to PHE. However, the positive peak around 800 cm^−1^ and the sharp positive band at ~1000 cm^−1^ can be assigned to AMA (Figure 4C). The other bands are mixed contributions from PVA and glycerol (Figure 4A,B).

PCA provides clear evidence that, in the data collected from films, the PHE contribution is not limited to the most intense feature at 1639 cm^−1^, but that the presence of the AI at different concentrations is identifiable across the full spectrum. Spectral unmixing approaches like NCLS can be performed using the full range, and the fit can be improved when multiple features from reference spectra of each compound can be identified in the film spectra.

### 3.4. Distribution and Content of PHE in PVA-GS-Based Films

**Figure 7:**
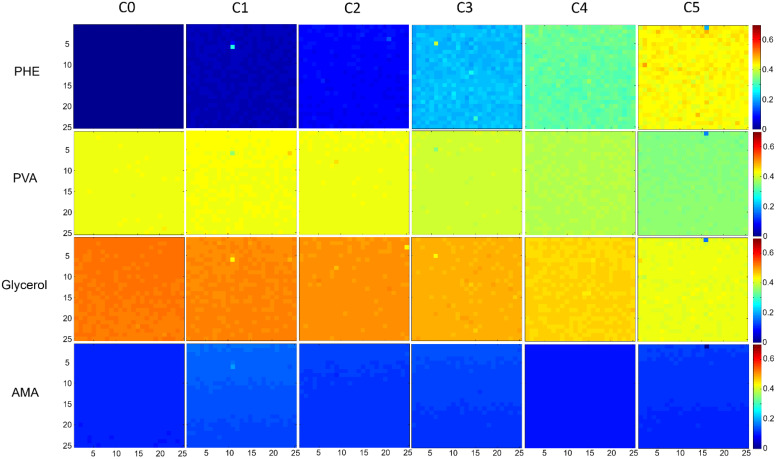
Typical reconstructed non-negatively constrained least squares (NCLS) maps following analysis of PVA-GS films loaded with increasing concentration of PHE: C0, C1, C2, C3, C4, and C5. The scale bar represents the contribution of each component spectrum in the Raman spectra at each pixel.

Figure 7 presents reconstructed false colour maps obtained from NCLS analysis applied to the first replicate (R1) of each PVA-GS film prepared for each concentration (C0 to C5). Rows represent the NCLS scores (spectral abundance fractions) calculated for PHE, PVA, Glycerol and AMA. A score equal to zero indicates no contribution for a given compound, while 1 corresponds to 100%, i.e., a spectrum from the map matching 100% with a pure spectrum from a reference. None of the images displayed NCLS scores higher than ~0.5–0.6, and therefore intensities are displayed between 0 and 0.7 for clarity and to help direct comparison of the reconstructed heat maps.

For C0, the NCLS analysis of Figure 7 correctly returns a 0% contribution for PHE, while the PVA, Glycerol, and AMA contributions are ~0.42, 0.53 and 0.10, respectively. The initial NCLS score for PHE for C0 (0.000394) is of great importance to verify that the model constructed using NCLS is reliable. It demonstrates there is no false positive, hence no overestimation in the determination of PHE in the films containing 0%. As previously discussed by Miloudi et al., the number of reference spectra used plays a key role in the specificity of the NCLS [26]. Notably, it is important that the reference spectra account for the matrix (i.e., PVA) in order to ensure that the algorithm builds the model on a robust basis to then estimate other constituents. For the purpose of this study, 1875 spectra collected from three PVA films were used in the NCLS.

Ratios between compounds are clearly seen to evolve systematically across the formulations C1–C5. While PHE maps display a change of colour from 0% contribution (deep blue) in C0 to ~45% in C5, reflecting the increase of concentrations in films, PVA and Glycerol have the opposite behaviour, with decreasing NCLS scores from C0 to C5. For AMA, the heat maps do not exhibit significant changes in intensity.

Irrespective of the PHE concentration tested, the NCLS reconstructed color-coded maps demonstrate the homogeneous distribution of the constituent components of the films. For concentration C5, the mean and standard deviations for NCLS scores are, respectively, 0.433 ± 0.014, 0.351 ± 0.009, 0.419 ± 0.011 and 0.112 ± 0.007 for PHE, PVA, Glycerol and AMA, corresponding to respective relative standard deviations (RSD) of 3.33%, 2.48%, 2.72% and 6.27%. These values confirm that the variations in concentration across the Raman maps of the dried films are minimal. The value, however, is slightly higher for AMA, suggesting a more heterogeneous distribution in the maps. Taking the mean from the three replicates, R1, R2 and R3, the RSD gives ranges from 0.58% (C1) to 1.74% (C5) for PVA and from 0.53% (C0) to 1.83% (C5) for Glycerol. For PHE, the mean RSD values are 4.90%, (C3), 4.43% (C4) and 3.70% (C5). For C0, C1 and C2, RSD values above 10% are reached, which can be explained by the low NCLS scores that progressively decrease towards 0, leading to increased weight of heterogeneity compared the concentration in the films. Nevertheless, it can be concluded that nanoformulation is an efficient way to ensure even distribution of a hydrophobic AI such as PHE in the hydrophilic matrix formed by polymers.

**Figure 8:**
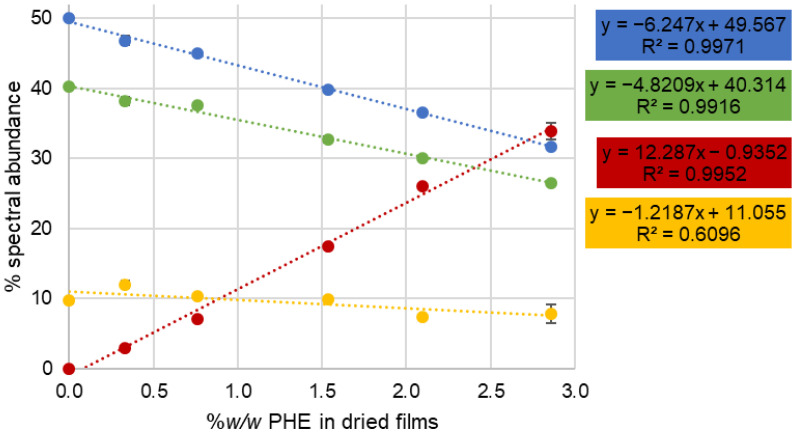
NCLS results with normalized abundance fraction of each component as a function of the concentration of PHE (%*w*/*w* in dried films). Red: PHE fraction, Blue: Glycerol fraction, Green: PVA fraction, and yellow: AMA fraction. Error bars represent standard error for *n* = 3 replicate films.

As regards estimating the respective content of ingredients in the films, NCLS scores cannot be used directly, as the relative spectral abundances are weighted by the respective spectral responses of each compound. Sums of spectral abundance exceed a value of 1, especially when the PHE concentration increases; hence, the correlation of NCLS results with actual constituent concentrations is not evident at first. For instance, in Figure 7 the sum of the NCLS scores corresponding to PHE, PVA, Glycerol and AMA for, respectively, C0, C1, C2, C3, C4 and C5 are 1.11, 1.14, 1.17, 1.26, 1.31 and 1.35. In this study, Raman analysis was performed on dry films; therefore, changing the amount of PHE inevitably modifies the concentration, expressed as %*w*/*w*, of all constituents. It is necessary to convert this notion to NCLS scores in order to have a better appreciation of the systematic evolution of the spectral abundance fractions calculated by NCLS as a function of PHE concentrations. Once the percentages of spectral abundance were calculated (see Material and Methods), it was possible to construct linear regression plots (Figure 8).

Raman response or Raman cross section dictates the relative strength of the signal originating from the compounds found in the analysed mixture. Depending on the chemical structures involved, the contribution of molecules can significantly differ from one to another. In the spectral map of C0, the relative spectral abundances of PVA and Glycerol are 0.42 and 0.53 respectively (PVA/Glycerol = 0.792). In Table 2, however, their nominal, as prepared %*w*/*w* contents are 46.30 and 48.96% (PVA/Glycerol = 0.946), indicating that the spectral contribution of PVA is relatively weaker than that of Glycerol by a factor of ~0.84. As can be seen from the linear regressions in Figure 8, this ratio of relative percentage of spectral abundance is maintained across the films as the concentration of PHE is increased. PVA and Glycerol have relative concentrations that are inversely correlated with PHE content, as also clearly observed in spectral abundance fractions in the NCLS reconstructed images (Figure 7, rows 2 and 3). In practice, Raman spectra are collected from a voxel defining the volume of analysis where the laser is focused. When analysis is performed on dry films, adding PHE inevitably leads to a decrease in PVA, Glycerol and AMA within this voxel. Linear regressions result in good fitting, with R^2^ = 0.9971 (Y = −6.247x + 49.567) and R^2^ = 0.9916 (Y = −4.8209x + 40.314), respectively, for Glycerol and PVA (Figure 8, blue and green). Similarly for AMA, taking into account the relative spectral abundance of 0.102 observed in image C0, it can be determined that its spectral contribution is ~2.4 stronger compared to PVA and ~2 stronger than Glycerol. AMA, however, has a lower R^2^ with 0.6096 (Y = −1.2187x + 11.055). The consistency of the NCLS analysis is evidenced by the fact that the regression slope for PHE, 12.287, is equal to the sum of the slopes for PVA, Glycerol and AMA (Figure 8).

The PHE percentage of spectral abundance of 0.0373% ± 0.008 for C0 increases to a maximum of 33.90 ± 1.21% for C5 (Figure 8, red). The linear regression obtained from the mean values of each concentration delivers a relationship of Y = 12.287x − 0.9352 with R^2^ = 0.9952, highlighting the proportionality of the Raman response according to the AI concentration in the dried films. However, while the concentration of PHE ranges from 0% *w*/*w* (C0) to 2.86% *w*/*w* (C5) (Table 2), the observed percent spectral abundance is ~12 times higher, suggesting the AI has a particularly strong Raman response compared to the other ingredients. This is confirmed by the spectral map of C1 (Figure 7), in which the relative spectral abundances of PHE and PVA are equal to 0.0334 and 0.427 (PHE/PVA = 0.0782), respectively. In Table 2, however, their nominal, as prepared %w/w contents are 0.33% and 47.2% (PHE/PVA = 0.00699), respectively, indicating that the spectral contribution of PHE is significantly stronger than that of PVA, by a factor of ~11.2. Similarly, in the spectral map of C1, the relative spectral abundance of Glycerol is 0.514, and hence PHE/Glycerol = 0.0650. In Table 3, however, its nominal, as prepared %*w*/*w* content is 47.5%, a ratio of PHE/Glycerol = 0.00694, indicating that the spectral contribution of PHE is also significantly stronger than that of Glycerol, by a factor of ~9.37.

The relative spectral abundance of AMA in the spectral map of C1 is 0.139, and hence PHE/AMA = 0.240 for a prepared concentration of 4.8% *w*/*w* (Table 2). The ratio PHE/AMA = 0.06875 suggest that the spectral contribution of PHE is also significantly stronger than that of AMA, by a factor of ~3.49.

### 3.5. Absolute Quantification of PHE in PVA-GS Films

**Figure 9:**
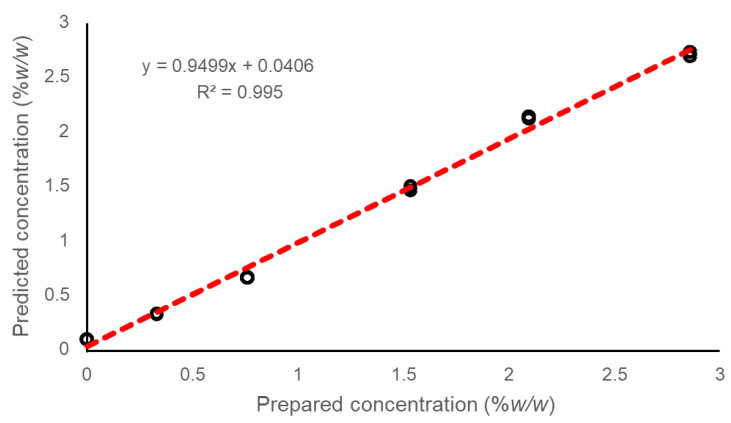
Absolute quantification of PHE in PVA-GS films. Prepared concentration %*w*/*w* regressed against predicted concentration %*w*/*w*.

NCLS analysis is an unmixing method that delivers results as scores, i.e., abundance fractions, for each compound introduced into the calculation for references. The expression of results as percentages of spectral abundance enables construction of a linear regression for PHE as a function of concentration in the film (Figure 8). Thanks to the proportionality between %*w*/*w* PHE in films and percent spectral abundance in the Raman data, a predictive model can be constructed. To demonstrate this, the map of R1 was used for calibration to define the equation of the linear regression (Y = 12.711x − 1.2796, R^2^ = 0.9939), and then the percent spectral abundance for all data points from maps R2 and R3 were determined as unknown samples. Figure 9 presents the prepared concentrations plotted against the predicted concentrations. This representation, widely used for spectral data subjected to quantitative data analysis [45,46,47,48], provides a visualisation of the quality of the fitting achieved. With Y = 0.9499x + 0.0406 and R^2^ = 0.995, the high correlation between the concentration of the AI in the dried films and the concentrations estimated using the NCLS results is confirmed. The RMSE is a standard way to measure the error of a model in predicting quantitative data. For instance, an RMSE = 0.0868% *w*/*w* PHE, that is, 6.07% of the median concentration of the range analysed, confirms the reliability of the analysis. The mean percent relative errors in the predicted concentration is equal to 3.75%, highlighting the accuracy of the analysis. Considering the concentrations individually, it is observed that the percentages of relative errors are, respectively, 1.61 ± 0.79, 13.6 ± 0.15, 3.42 ± 1.81 and 1.92 ± 0.70 for C1, C2, C3, C4 and C5. Except for C2, the concentrations are predicted with relative errors below 5%.

NCLS analysis of Raman microspectroscopic profiles can therefore be employed to perform quantitative analysis of the percentage of AI content within a composite solid film matrix with a high degree of accuracy. The methodology of mapping provides a qualitative analysis of the film in terms of, for example, the degree of homogeneity of the composition, and also provides relatively large datasets to construct and validate calibration models for absolute quantitation purposes.

## 4. Conclusions

First, chemical maps reconstructed from Raman maps using NCLS confirm the homogeneous distribution of PHE, the active ingredient, after drying. The results support stabilisation of hydrophobic active cosmetic ingredients such as Pluronic^®^ F127 stabilised nanoformulation as a promising approach to prepare film-forming cosmetics for topical application. In addition, NCLS analysis enables the distribution of other ingredients to be visualised, specifically thanks to a collection of reference Raman spectra collected from pure compounds. Linear regressions for PVA, Glycerol and PHE displayed R^2^ > 0.99, highlighting their high correlation in Raman features with varying amounts of the AI within the films. Ultimately, it is further demonstrated that NCLS scores, i.e., calculated spectral abundance for each ingredient, can be used to perform absolute quantification. The R^2^ = 0.995 between the %*w*/*w* prepared and %*w*/*w* predicted concentrations strengthens the potential of Raman spectroscopy coupled to multivariate analysis as a tool to support the development of new film-forming topical products. Second, this study demonstrates that confocal Raman Imaging is a valuable non-destructive, label-free and reagent-free tool that can support the development of green analytical protocols in the cosmetics industry. In addition to delivering spatially resolved chemical maps with micrometric resolution, this technique can contribute greatly to reducing the consumption of consumables and solvents from the early stages of development of new formulae to final quality control of products. Coupling Raman confocal imaging with advanced multivariate analysis such as NCLS opens perspectives for standardisation and automation of data mining protocols, and thus for its implementation for daily use in the industry.

## Figures and Tables

**Table 1:** Quantity of PVA solution, Glycerol, AMA, and NanoPHE for the preparation of film-forming solutions with a range of five concentrations of NanoPHE.

N°	PVA Solution (g)	Glycerol (g)	AMA (g)	NanoPHE (g)
C0	8.8065	1.0572	0.1006	- (0.1162 g of water)
C1	8.80850	1.0140	0.1027	0.11500
C2	8.65000	0.9957	0.1023	0.26190
C3	8.40000	1.0020	0.1010	0.53590
C4	8.15000	1.0147	0.1017	0.74240
C5	7.89930	1.0031	0.1020	1.02180

**Table 2:** Concentration of PHE, PVA, Glycerol and AMA in dried film (%*w*/*w*).

N°	Nominal % of PHE (*w*/*w*)	Nominal % of PVA (*w*/*w*)	Nominal % of Glycerol (*w*/*w*)	Nominal % of AMA (*w*/*w*)
C0	0	46.3	48.96	4.7
C1	0.333	47.2	47.5	4.8
C2	0.761	47.1	46.9	4.8
C3	1.53	46.5	46.5	4.7
C4	2.09	45.7	46.4	4.6
C5	2.86	45.5	45.5	4.6

**Table 3:** Raman shift assignments for PHE spectrum.

Raman Shift (cm^−1^)	Shift Assignments
1636–1639	C=C stretching
1440	CH_2_ scissoring, asymmetric CH_3_ bending
1294	C-OH (enol group) bending, C-C-H bending (aromatic ring), CH_2_ twisting
1257	C-C stretching, =CH bending
1202	C-C stretching
1169	C-O stretching, C-C stretching
1114	C-O stretching, C-C stretching
846	C-C stretching

## Data Availability

The data presented in this study are available on request from the corresponding author.

## Sample Availability

Samples of the compounds are not available from the authors.

## References

[1] Voss G.T., Gularte M.S., de Oliveira R.L., Luchese C., Fajardo A.R., Wilhelm E.A. (2020). Biopolymeric Films as Delivery Vehicles for Controlled Release of Hydrocortisone: Promising Devices to Treat Chronic Skin Diseases. Mater. Sci. Eng. C.

[2] Kathe K., Kathpalia H. (2017). Film Forming Systems for Topical and Transdermal Drug Delivery. Asian J. Pharm. Sci..

[3] Pünnel L.C., Lunter D.J. (2021). Film-Forming Systems for Dermal Drug Delivery. Pharmaceutics.

[4] Frederiksen K., Guy R.H., Petersson K. (2016). The Potential of Polymeric Film-Forming Systems as Sustained Delivery Platforms for Topical Drugs. Expert Opin. Drug Deliv..

[5] Rao P.R., Diwan P.V. (1998). Formulation and in Vitro Evaluation of Polymeric Films of Diltiazem Hydrochloride and Indomethacin for Transdermal Administration. Drug Dev. Ind. Pharm..

[6] Zurdo Schroeder I., Franke P., Schaefer U.F., Lehr C.-M. (2007). Development and Characterization of Film Forming Polymeric Solutions for Skin Drug Delivery. Eur. J. Pharm. Biopharm..

[7] Frederiksen K., Guy R.H., Petersson K. (2015). Formulation Considerations in the Design of Topical, Polymeric Film-Forming Systems for Sustained Drug Delivery to the Skin. Eur. J. Pharm. Biopharm..

[8] Mandapalli P.K., Labala S., Jose A., Bhatnagar S., Janupally R., Sriram D., Venuganti V.V.K. (2017). Layer-by-Layer Thin Films for Co-Delivery of TGF-β SiRNA and Epidermal Growth Factor to Improve Excisional Wound Healing. AAPS PharmSciTech.

[9] Aisyah Y., Irwanda L.P., Haryani S., Safriani N. (2018). Characterization of Corn Starch-Based Edible Film Incorporated with Nutmeg Oil Nanoemulsion. IOP Conf. Ser. Mater. Sci. Eng..

[10] Alkilani A., McCrudden M.T., Donnelly R. (2015). Transdermal Drug Delivery: Innovative Pharmaceutical Developments Based on Disruption of the Barrier Properties of the Stratum Corneum. Pharmaceutics.

[11] Amnuaikit C., Ikeuchi I., Ogawara K., Higaki K., Kimura T. (2005). Skin Permeation of Propranolol from Polymeric Film Containing Terpene Enhancers for Transdermal Use. Int. J. Pharm..

[12] The Mystical Effects of Dermatological Vehicles-FullText-Dermatology 2005, Vol. 210, No. 2-Karger Publishers. https://www.karger.com/Article/FullText/82572.

[13] Adrover A., di Muzio L., Trilli J., Brandelli C., Paolicelli P., Petralito S., Casadei M.A. (2020). Enhanced Loading Efficiency and Mucoadhesion Properties of Gellan Gum Thin Films by Complexation with Hydroxypropyl-β-Cyclodextrin. Pharmaceutics.

[14] Munnier E., Al Assaad A., David S., Mahut F., Vayer M., Van Gheluwe L., Yvergnaux F., Sinturel C., Soucé M., Chourpa I. (2020). Homogeneous Distribution of Fatty Ester-based Active Cosmetic Ingredients in Hydrophilic Thin Films by Means of Nanodispersion. Int. J. Cosmet. Sci..

[15] Contardi M., Heredia-Guerrero J.A., Perotto G., Valentini P., Pompa P.P., Spanò R., Goldoni L., Bertorelli R., Athanassiou A., Bayer I.S. (2017). Transparent Ciprofloxacin-Povidone Antibiotic Films and Nanofiber Mats as Potential Skin and Wound Care Dressings. Eur. J. Pharm. Sci..

[16] Patel S., Srivastava S., Singh M.R., Singh D. (2018). Preparation and Optimization of Chitosan-Gelatin Films for Sustained Delivery of Lupeol for Wound Healing. Int. J. Biol. Macromol..

[17] Gehrcke M., de Bastos Brum T., da Rosa L.S., Ilha B.D., Soares F.Z.M., Cruz L. (2021). Incorporation of Nanocapsules into Gellan Gum Films: A Strategy to Improve the Stability and Prolong the Cutaneous Release of Silibinin. Mater. Sci. Eng. C.

[18] Niamlang P., Tongrain T., Ekabutr P., Chuysinuan P., Supaphol P. (2017). Preparation, Characterization and Biocompatibility of Poly(Vinyl Alcohol) Films Containing Tetracycline Hydrochloride-Loaded Quaternized Chitosan Nanoparticles. J. Drug Deliv. Sci. Technol..

[19] Magalhães T.M., Guerra R.C., da San Gil R.A.S., Valente A.P., Simão R.A., Soares B.G., de Carvalho Mendes T., dos Santos Pyrrho A., de Sousa V.P., Rodrigues-Furtado V.L. (2017). PAMAM Dendrimer Hydrogel Film—Biocompatible Material to an Efficient Dermal Delivery of Drugs. J. Nanopart. Res..

[20] Garvie-Cook H., Frederiksen K., Petersson K., Guy R.H., Gordeev S. (2015). Characterization of Topical Film-Forming Systems Using Atomic Force Microscopy and Raman Microspectroscopy. Mol. Pharm..

[21] Schmidt U., Hild S., Ibach W., Hollricher O. (2005). Characterization of Thin Polymer Films on the Nanometer Scale with Confocal Raman AFM. Macromol. Symp..

[22] Ghita A., Pascut F.C., Sottile V., Denning C., Notingher I. (2015). Applications of Raman Micro-Spectroscopy to Stem Cell Technology: Label-Free Molecular Discrimination and Monitoring Cell Differentiation. EPJ Tech. Instrum..

[23] Rammal H., Al Assaad A., Dosio F., Stella B., Maksimenko A., Mura S., Van Gulick L., Callewaert M., Desmaële D., Couvreur P. (2021). Investigation of Squalene-Doxorubicin Distribution and Interactions within Single Cancer Cell Using Raman Microspectroscopy. Nanomed. Nanotechnol. Biol. Med..

[24] Dorney J., Bonnier F., Garcia A., Casey A., Chambers G., Byrne H.J. (2012). Identifying and Localizing Intracellular Nanoparticles Using Raman Spectroscopy. Analyst.

[25] Beleites C., Bonifacio A., Codrich D., Krafft C., Sergo V. (2013). Raman Spectroscopy and Imaging: Promising Optical Diagnostic Tools in Pediatrics. CMC.

[26] Miloudi L., Bonnier F., Tfayli A., Yvergnaux F., Byrne H.J., Chourpa I., Munnier E. (2018). Confocal Raman Spectroscopic Imaging for in Vitro Monitoring of Active Ingredient Penetration and Distribution in Reconstructed Human Epidermis Model. J. Biophotonics.

[27] Paudel A., Raijada D., Rantanen J. (2015). Raman Spectroscopy in Pharmaceutical Product Design. Adv. Drug Deliv. Rev..

[28] Waffo Tchounga C.A., Sacre P.Y., Ciza P., Ngono R., Ziemons E., Hubert P., Marini R.D. (2021). Composition Analysis of Falsified Chloroquine Phosphate Samples Seized during the COVID-19 Pandemic. J. Pharm. Biomed. Anal..

[29] Miloudi L., Bonnier F., Bertrand D., Byrne H.J., Perse X., Chourpa I., Munnier E. (2017). Quantitative Analysis of Curcumin-Loaded Alginate Nanocarriers in Hydrogels Using Raman and Attenuated Total Reflection Infrared Spectroscopy. Anal. Bioanal. Chem..

[30] Mazurek S., Szostak R. (2016). Quantitative Analysis of Topical Gels and Ointments by FT-Raman Spectroscopy. Vib. Spectrosc..

[31] Strachan C.J., Rades T., Gordon K.C., Rantanen J. (2010). Raman Spectroscopy for Quantitative Analysis of Pharmaceutical Solids. J. Pharm. Pharmacol..

[32] Hertrampf A., Sousa R.M., Menezes J.C., Herdling T. (2016). Semi-Quantitative Prediction of a Multiple API Solid Dosage Form with a Combination of Vibrational Spectroscopy Methods. J. Pharm. Biomed. Anal..

[33] Bakkar M.A., Nawaz H., Majeed M.I., Naseem A., Ditta A., Rashid N., Ali S., Bajwa J., Bashir S., Ahmad S. (2021). Raman Spectroscopy for the Qualitative and Quantitative Analysis of Solid Dosage Forms of Sitagliptin. Spectrochim. Acta Part A Mol. Biomol. Spectrosc..

[34] Bajwa J., Nawaz H., Majeed M.I., Hussain A.I., Farooq S., Rashid N., Bakkar M.A., Ahmad S., Hyat H., Bashir S. (2020). Quantitative Analysis of Solid Dosage Forms of Cefixime Using Raman Spectroscopy. Spectrochim. Acta Part A Mol. Biomol. Spectrosc..

[35] Kwan C., Ayhan B., Chen G., Wang J., Ji B., Chang C.-I. (2006). A Novel Approach for Spectral Unmixing, Classification, and Concentration Estimation of Chemical and Biological Agents. IEEE Trans. Geosci. Remote Sens..

[36] Chiper M., Hervé Aubert K., Augé A., Fouquenet J.-F., Soucé M., Chourpa I. (2013). Colloidal Stability and Thermo-Responsive Properties of Iron Oxide Nanoparticles Coated with Polymers: Advantages of Pluronic^®^ F68–PEG Mixture. Nanotechnology.

[37] Gupta S., Gupta S., Jindal N., Jindal A., Bansal R. (2013). Nanocarriers and Nanoparticles for Skin Care and Dermatological Treatments. Indian Dermatol. Online J..

[38] Guo C., Liu H., Wang J., Chen J. (1999). Conformational Structure of Triblock Copolymers by FT-Raman and FTIR Spectroscopy. J. Colloid Interface Sci..

[39] Beattie J.R., Maguire C., Gilchrist S., Barrett L.J., Cross C.E., Possmayer F., Ennis M., Elborn J.S., Curry W.J., McGarvey J.J. (2007). The Use of Raman Microscopy to Determine and Localize Vitamin E in Biological Samples. FASEB J..

[40] Wang K., Sun D.-W., Wei Q., Pu H. (2018). Quantification and Visualization of α-Tocopherol in Oil-in-Water Emulsion Based Delivery Systems by Raman Microspectroscopy. LWT.

[41] Yang C.-C., Chiu S.-J., Lee K.-T., Chien W.-C., Lin C.-T., Huang C.-A. (2008). Study of Poly(Vinyl Alcohol)/Titanium Oxide Composite Polymer Membranes and Their Application on Alkaline Direct Alcohol Fuel Cell. J. Power Sources.

[42] Mudalige A., Pemberton J.E. (2007). Raman Spectroscopy of Glycerol/D2O Solutions. Vib. Spectrosc..

[43] Badawi H.M. (2011). Vibrational Spectra and Assignments of 2-Phenylethanol and 2-Phenoxyethanol. Spectrochim. Acta Part A Mol. Biomol. Spectrosc..

[44] Ansari Z., Bhattacharya T.S., Saha A., Sen K. (2018). Block Copolymer Mediated Generation of Bimetallic Ni-Pd Nanoparticles: Raman Sensors of Ethyl Paraben and Ciprofloxacin. React. Funct. Polym..

[45] Elderderi S., Wils L., Leman-Loubière C., Henry S., Byrne H.J., Chourpa I., Munnier E., Elbashir A.A., Boudesocque-Delaye L., Bonnier F. (2021). Comparison of Raman and Attenuated Total Reflectance (ATR) Infrared Spectroscopy for Water Quantification in Natural Deep Eutectic Solvent. Anal. Bioanal. Chem..

[46] Elderderi S., Wils L., Leman-Loubière C., Byrne H.J., Chourpa I., Enguehard-Gueiffier C., Munnier E., Elbashir A.A., Boudesocque-Delaye L., Bonnier F. (2021). In Situ Water Quantification in Natural Deep Eutectic Solvents Using Portable Raman Spectroscopy. Molecules.

[47] Makki A.A., Elderderi S., Massot V., Respaud R., Byrne H.J., Tauber C., Bertrand D., Mohammed E., Chourpa I., Bonnier F. (2021). In Situ Analytical Quality Control of Chemotherapeutic Solutions in Infusion Bags by Raman Spectroscopy. Talanta.

[48] Makki A.A., Massot V., Byrne H.J., Respaud R., Bertrand D., Mohammed E., Chourpa I., Bonnier F. (2021). Vibrational Spectroscopy for Discrimination and Quantification of Clinical Chemotherapeutic Preparations. Vib. Spectrosc..

